# Internet-Based Survey on Physical Activity and Incidence of Injury in Active Working Dogs

**DOI:** 10.3390/ani13101647

**Published:** 2023-05-16

**Authors:** Giuseppe Spinella, Simona Valentini, Mirella Lopedote

**Affiliations:** 1 Department of Veterinary Medical Sciences, University of Bologna, Ozzano dell’Emilia, 40064 Bologna, Italy; simona.valentini@unibo.it; 2 Ambulatorio Veterinario Fisio & Sport, Grumo di San Michele all’Adige, 38010 Trento, Italy; mirella.lopedote@yahoo.com

**Keywords:** working dogs, survey, injury, clinical management, sports examination, orthopedic examination, warm-up, cool-down

## Abstract

**Simple Summary:**

One hundred and nine respondents, to a survey on medical and preventive management of working dogs, reported a relatively low percentage of dogs submitted to a specific sports medical examination (36.4%) or to a generic orthopaedic examination (55.5%) before starting their professional activity (conditioning and competition). Injury incidence was relatively high (45.5%), even if mostly represented by mild muscular trauma; and preventive exercise or pre- and post-activity exercises (warm-up and cool-down phases) were performed sporadically.

**Abstract:**

A survey with 100 multiple choice and open-ended questions was proposed by free access to working dogs’ handlers. One hundred and nine respondents were recorded and their dates processed. The most represented breeds were: Belgian Malinois, Labrador, Border Collie and German Shepherds. Of these, 71.6% were intact dogs and 28.4% were spayed or neutered, with a median age range of 3–4 years. Furthermore, 55.5% had undergone early radiographic examinations for hip or elbow dysplasia diagnosis. The dogs performed the following activities: search and rescue on surface (59%), search and rescue on rubble (37%), Internationale Gebrauchshund Pruefung (IGP) (9%), man trailing (5%), sled dog (5%), search on avalanche (4%), dog towing (3%), canine shows (3%), hunting (2%), water rescue (1%), pet therapy (1%), wildlife conservation dog (1%), Mondioring (1%). Only 36.4% of respondents submitted their dogs to a specific sports medical examination and 55.5% to an orthopaedic examination. An injury incidence of 45.5% was recorded, generally related to mild musculoskeletal trauma. A limited number of handlers routinely performed warm-up and/or cool-down activities. A positive assessment emerged of the need for many respondents to attend and request education courses and updates on the proper health management of their dogs.

## 1. Introduction

Presently, working dogs are actively involved in specific and professional tasks aimed to improve civil security and disaster risk management. The role of the dog is essential to allow a greater degree of safety for human health, especially when natural disasters or missing persons occur.

The relationship established between the dog and the handler is incredibly symbiotic and the handler experience represents a good indicator and an essential source of information regarding the possibility of receiving useful data to improve the therapeutic approach and in the prevention of common musculoskeletal pathologies [1,2,3,4,5].

In sports medicine, injury incidence seems to be relatively higher in dogs performing high-impact sports, such as agility and flyball. The literature on agility dogs has reported that 32–41.7% had experienced at least one injury, most commonly located at the shoulder region, iliopsoas muscle, back region, and digits [1,3,4]. Moreover, Border Collies are significantly overexpressed as a breed more predisposed to trauma and the rate of dogs that sustained at least one injury varied by country, depending on national regulations and soil characteristics, with the highest percentage reported in Australia and the lowest percentage reported in the US [1]. No relationship was found between the use of warm-up and cool-down exercises and injury, although it is a commonly held belief in the canine agility community that these exercises are important factors in reducing the risk of injury in dogs [6]. Unfortunately, evidence from the literature on the effects of warm-up and cool-down exercises remains inconclusive. A systematic review of injuries in human athletes found equivocal results regarding whether these factors can modify the risk of injury [7].

Injuries in dogs performing flyball activity reported a similar rate (34–39%) compared with agility [8,9,10].

Currently, the specialist literature does not report a high number of musculoskeletal injuries in working dogs engaged in humanitarian missions or in environmental disasters [11]. Pathologies reported in working dogs are generally connected to the environmental conditions (dehydration, diarrhoea, vomiting, etc.) or to the characteristics of the surfaces on which they were employed during their ‘professional’ activity (burns, gunshot wounds, sliding lesions, etc.) [12]. On the other hand, veterinary management and the incidence of trauma in working dogs’ patent trials and training activities have not been widely investigated.

Generally, when injuries occur and veterinary personnel are not available, it has been reported that 40–50% of healthcare providers (HCPs) were enrolled in the Joint Forces Combat Trauma Management Course for managing emergently injured military working dogs. Care provided by HCPs is limited to circumstances in which the dog is too unstable to be transported to veterinary facilities or medical evacuation is not possible; immediate care is necessary to preserve life, limb or eyesight [13].

The aim of this study was to describe the frequency and types of injuries experienced by working dogs, also investigate their healthcare management, nutrition and training activities. This investigation was conducted through an internet survey of working dogs’ handlers.

On the basis of the author’s clinical experience and caseload, we hypothesised that the prevalence of injuries would be lesser than high-impact sports dogs (agility and flyball).

## 2. Materials and Methods

An online survey (anonymous self-administered form) was advertised through clinical newsletters, social media (Messenger), farm groups and working dog associations. Owners could address the survey only once for each owned dog and only questionaries that received the consent for publication by owners were included in this study. The questionnaire consisted of 100 questions (multiple choice and open-ended questions) and was subdivided into 11 main sections according to the following complaints: 1. signalment and current health status; 2. dog diet; 3. dietary supplements or medication; 4. dogs’ housing; 5. transport; 6. training tools; 7. work the dogs were trained to perform; 8. training protocol; 9. injury prevention; 10. location, diagnosis and treatment of injuries; 11. owner training and education (Supplementary Materials). Some questions were optional for owners, whereas other answers were mandatory in order to continue with the questionnaire. Once the survey was completed, none of the answers could be changed. A pie chart of the percentages obtained by multiple choice questions was automatically provided: otherwise, the authors could access the individual questionnaires to view each individual response. In the open-ended questions, the individual answers were read and recorded by the researchers.

The questionnaire was also associated with informed consent (Supplementary Section 12) to data processing and further publication in an anonymous form, which was requested before the survey closure. Moreover, an optional Supplementary Section 13 was dedicated to free suggestions and comments on working dogs’ welfare (Supplementary Materials).

## 3. Results

Data collection was carried out in a five-month period, from January to May 2022: a total of 109 respondents participated in the study by completing the survey (answering all mandatory questions).

SECTION 1—Signalment and current health status—The breeds included in the study were Belgian Malinois (n = 17), Labrador Retriever (n = 17), Border Collie (n = 17), German Shepherd (n = 12), Golden Retriever (n = 9), mixed breed (n = 7), Australian Shepherd Kelpie (n = 5), Siberian Husky (n = 5), Dutch Shepherd (n = 2), Czechoslovakian Wolfdog (n = 2), Dog Beauceron Shepherd (n = 2), Langhaar (n = 1), Dobermann (n = 1), Red Irish Setter (n = 1), Giant Schnauzer (n = 1), Weimaraner (n = 1), German Pinscher (n = 1), American Akita (n = 1), Lakeland Terrier (n = 1), Cane Corso (n = 1), German Longhaired Pointer (n = 1), Hovawart (n = 1), Italian Hound (n = 1), Giant Poodle (n = 1), Pointer (n = 1). Out of 109 dogs considered, 71.6% were breeding dogs and 28.4% were spayed/neutered. The age of the dogs was: <1 year (n = 7), 1–2 years (n = 21), 3–4 years (n = 29), 5–6 years (n = 21), 7–8 (n = 20), 9–11 years (n = 11). In questions 4–10, the prophylaxis against the main pathologies carried by vectors and external parasites was taken into consideration. In particular, the deworming treatment rate was 25.4% as a puppy, sporadically in 16.4%, once a month in 20%, once a year in 38.2%. Heartworm prevention was as follows: 10% never, 3.6% sporadically, 41.7% once a month, 43.6% once a year and 2% unspecified. Tablets (62.4%), vaccination (28.7%), spot on (4.45%) or other unspecified methods (4.45%) were used for prophylaxis. Leishmaniasis prevention was carried out as follows: never (14.5%), sporadically (7.7%), once a month (28.2%), once a year (50%); and collar (33.7%), vaccination (38%), spot on (28.3%) were used for prophylaxis. Control of external parasites was carried out as follows: 3.7% never, 7.3% sporadically, 64.5% once a month, 24.5% once a year; collar (30.5%), spot on (33.3%), tablets (30.5%) and other unspecified forms (5.7%) were used for prophylaxis.

Next, the interviewees were asked if their dogs had undergone early and/or ultimate diagnostic radiographic examination (for example for hip or elbow dysplasia) and which anatomical districts were investigated. Of these, 55.5% of the dogs underwent early radiographic examinations whereas 44.5% did not.

Early radiographic examinations were carried out on hips (n = 45), elbows (n = 36), knees (n = 1), shoulders (n = 2); confirmatory radiographic tests were performed on elbows (n = 44), hips (n = 52), shoulders (n = 5), spine (n = 2).

Overall, 55.5% of the dogs were submitted to an orthopaedic examination, 43.6% were not and 0.9% had an orthopaedic evaluation during standard radiographic assessment. The age at which the orthopaedic examination was performed was three months (n = 2), four months (n = 1), five months (n = 5), six months (n = 8), seven months (n = 2), eight months (n = 1), one year (n = 14), two years (n = 12), three years (n = 4), four years (n = 4), five years (n = 2), six years (n = 5), seven years (n = 1). The reasons for visiting were routine clinical examination (n = 35) and suspected muscular-skeletal diseases (n = 9).

As working dogs generally require high performance, we assessed whether the dog had ever undergone a specific sports medical examination. One hundred and nine responses were divided as follows: 63.6% never, 36.4% yes. Fifteen dogs had routinely undergone a sports medical exam (every year).

Overall, 39.1% of dogs had undergone general blood tests once a year; 9.1% only as a puppy; 31.8% sporadically and 20% never.

It was also asked whether and why the dog had ever undergone to a cardiological examination. To this, 62.7% answered that they had never undergone a cardiological examination, 19.1% once a year, 11.8% sporadically, and 6.4% only as a puppy.

Thirty-two owners did not know the physiological parameters of their dogs, 20 owners relied on their veterinarian to evaluate these parameters and 28 owners knew both the parameters and the range of values.

SECTION 2—Dog diet—Of the interviewees, 79.1% administered dry commercial food, 10.9% BARF, 3% cooked meat, 2% commercial wet food, 5% other; 83.5% give two meals a day, 10.1% three meals, 6.4% one meal a day. Responses to fasting times before work were highly variable, ranging from 2 to 12 h. Furthermore, 53.2% of the interviewees did not make any food changes during the year while 46.8% changed feeding.

SECTION 3—Dietary supplements or medication—Of the interviewees, 50.9% did not administer any type of supplement, 49.1% administered supplements, above all multivitamin complexes (25.9%) and omega-3 supplements (22.2%), two–three cycles of treatment per year. When asked about the administration of chondroprotectors, 50.1% gave them quarterly. Overall, 94.5% of the dogs were not on medication, while 5.5% of owners gave an affirmative answer, but then when asked what kind of drug the dog took, they referred to the medication, not the specific problem and the medications were highly variable.

SECTION 4—Dogs’ housing—Questions were asked about the domestic environment where the dog usually lived in order to examine the daily habits and whether the domestic environment could influence the activity carried out by the dog, injury occurrence and the relationship with the handler. Overall, 57.3% of dogs lived between the owner’s home and garden, 36.4% exclusively in the home, 4.3% exclusively in the garden and 2% in a place other than the handler’s home. For dogs living in the garden, 60% lived free, while 40% were fenced. The surface on which the dogs spent most of their time was grass (60%) house tiles (30%), gravel and soil (10%). Additionally, 53.6% of dogs slept on a ‘dog bed’, 12.7% slept in kennels, 10.9% on the floor, 22.8% on other types of unspecified materials.

SECTION 5—Transport—Overall, 90% of dogs are transported by car: 89.1% in a cage, 8.2% unrestrained and 2.7% restrained, while 10% of dogs are transported in van, always in special kennels. The environment in which the dog travelled was usually air-conditioned (86.4%), but not in 13.6% of cases. The journey had a variable duration, ranging from a minimum of 10 min to a maximum of six hours, with an average value of about 1.5 h. Furthermore, 20.9% of the dogs, due to their specific work, required other types of transport to reach the search field; 14.3% are ferried, 80% are transported by helicopter and 5.7% by cable car.

SECTION 6—Training tools—When the dog was not working, in 83.6% of cases it usually wore a collar or harness. Dogs were restrained with a leash whose length varied from 1 to 3 metres (52.7%), 39.1% of the dogs were unrestrained and 8.2% were restrained with other techniques. During work, 51.8% of dogs wore a specific harness: a rescue harness (80%), a helicopter harness, a sledding harness, a utility and tactical harness, a climbing and tracking harness. Additionally, 48.2% of dogs wore nothing while working. Eighty percent of handlers who used a specific harness took them off after work, while the remaining 20% did not remove them. Evaluating the reaching of inaccessible search places via helicopter rescue, it was asked if the handlers noticed a difference in the dog working after removing the harness: 100% of them did not show any discrepancy.

SECTION 7—Work the dogs were trained to perform—The dogs performed the following work/activities: search on surface (59%), search on rubble (37%), Internationale Gebrauchshund Pruefung (9%), man trailing (5%), sled dog (5%), search on avalanche (4%), dog towing (3%), canine shows (3%), hunting (2%), water rescue (1%), pet therapy (1%), wildlife conservation dog (1%), Mondioring (1%). The dogs’ work was part-time (78.2%) or full-time (21.8%): dogs started working at ages ranging from 2 to 15 months. Dogs worked three times a week (32%), twice a week (28.5%), four days a week (12%), every day (15%) and only 8% one day a week; 4.5% of owners did not provide a clear answer. Forty-eight percent were in activity, i.e., they had passed exams so in the event of natural disasters or missing people they could be activated by civil protection and work in the field. Overall, 34.6% of dogs were still in training, whereas 7.4% had passed aptitude exams, i.e., they have passed the training phase and were preparing for the operational phase; the remaining 10% worked sporadically. The dogs worked on different ground surfaces: 80% woodland and the rest consisting of rubble, snow, the aquatic environment and grass.

SECTION 8—Training protocol—Only 5% of the owners answered correctly to the question ‘In your opinion, what is a training program’, identifying as the purpose of the training program the physical fitness, that included cardiorespiratory (i.e., aerobic and anaerobic capacity) and neuromuscular (i.e., muscle strength, mobility, balance) components [5]. However, 95% of the owners described the weekly training in detail. Most handlers described obedience exercises as training, others trained the dog with mountain runs, swimming or walks, with an average of three times a week. Only 15% of the dogs carried out specific training for their work.

SECTION 9—Injury prevention—Overall, 42.7% of handlers carried out prevention techniques, i.e., massage (17%), warm-up (22%), stretching (9%), cool-down (11%) and proprioception exercises (2%). When asked about the type of warm-up carried out before a work session, 80% did not warm-up properly, describing only a run with ball throwing, whereas 20% described a walk of about five minutes. With regard to the cool-down, after working, the dogs rested directly in the kennel (80%), took a walk (15%), and did stretching exercises and massages (5%). The prevention protocol was drafted by the owners autonomously (45.5%), by the physiatrist vet (17.5%), by the coach (12.5%), by the vet (4.5%), by other unspecified figures (20%). Twenty-eight owners answered that they did nothing to prevent limb injuries, two repliers performed massage, three maintained a healthy weight, five performed muscle strengthening exercises, four handlers practiced proprioception exercises on their dogs, eight avoided jumping when they realised that the dog was not fit, ten owners performed warm-up; 90% did nothing to prevent spine injuries, the remaining 10% massaged and warmed up and avoided jumping. Regarding time dedicated to prevention, 90% of 109 respondents answered negatively (i.e., they did not dedicate time to prevention), 10% dedicated just a few minutes a week, with activities mainly represented by walks or massages (37.7%).

SECTION 10—Location, diagnosis and treatment of injuries—Of the owners, 80% identified pain with lameness, 20% with both behavioural changes and inappetence. Overall, 54.5% of dogs have never suffered an injury, whereas 45.5% had reported an injury, i.e., muscle injuries (22%), phalangeal or carpal fractures (20%), fingertip lesions (10%), cruciate ligament rupture (3%) and shoulder injuries (3%). In 40% of cases, the symptoms occurred in the days following the trauma, in 60% on the same day. In 91.8% of cases, the injury was temporary and never recurred, in 8.2% it was permanent. The time range of rest for the dog varied between seven days and three months depending on the type of injury found. The diagnosis was made in 79.1% of cases by a veterinary surgeon, and in 20.9% independently by the owner. Post-traumatic treatment was decided by the veterinary surgeon (70%), autonomously by the owner (28.2%) or by the coach (1.8%). Only 20.9% of dogs required physiotherapy. Out of 37 responses from those who used physiotherapy, 90% of the dogs were treated with laser therapy and underwater treadmill, and 10% with anti-inflammatories. About 10% of the dogs required surgical treatment, but not all respondents stated which specific surgery was performed.

SECTION 11—Owner training and education—Of the owners, 42.7% followed courses on the well-being of their dog, above all courses held by veterinary surgeons or pharmaceutical companies. Of these, 83.6% agreed to submit their dogs to a specific sports medical visit; 16.4% do not consider it necessary.

## 4. Discussion

The aim of this survey was to obtain information on injuries experienced by working dogs, and also investigate their healthcare management, nutrition and training activities. We hypothesised that the prevalence of injuries would be lesser than high-impact sports dogs, but on the basis of our sample, the hypothesis was not confirmed, as handlers reported a 45.5% injury incidence. Moreover, the outcomes of our survey highlighted some rather questionable aspects in relation to canine health management, the early radiological screening of congenital articular pathologies that could be a source of pain and limitation for work activity (hip or elbow dysplasia) and finally, for injury prevention.

The first section of our survey was dedicated to canine signalment and clinical management. Belgian Malinois, Labradors and German Shepherds were the breeds more expressed, in line with previous scientific studies on working dogs; with similar results also for the age of dogs routinely involved in activities of search and rescue and other civil security and disaster risk management [5,11,14]. A different distribution was observed for gender status, with a higher percentage of intact dogs. This could be a consequence of the higher percentage of Labrador and Golden Retrievers included in this study and dogs are generally maintained sexually intact in order to retain the better subjects for breeding. However, the condition of intact males and females has been correlated to a significant increase of hip dysplasia, cranial cruciate ligament tear and elbow dysplasia; moreover, in male Golden and Labrador Retriever dogs, the neutering status has shown a decreased neoplasia occurrence incidence; whereas, in female Golden Retrievers neutering increased the rate of neoplasia [15]. In agility dogs, the neutered status was correlated to a higher risk of injury: this was in contrast with our sample, as neutered dogs reported a similar incidence of injuries compared with intact dogs [16].

Vaccination for disease prevention is mandatory for all working and athlete dogs, especially for working dogs that act in contact with several other teams of dogs and are often transported in extremely varied environments or foreign countries [17].

The final part of the first section was dedicated to the clinical examination of dogs specifically selected for working activities, with particular attention to early radiological screening in order to verify the initial joint conditions and exclude dogs potentially affected by hip or elbow dysplasia, both pathologies that could provide structural instability generating pain during work [17]. In our opinion, it is surprising that only 55.5% of owners had submitted their dogs to an early radiological screening, considering that most working dogs start training at six to eight months of age and that pathologies such as hip or elbow dysplasia could be a source of pain and limitation of activity during conditioning. Moreover, sanitary management should be focused not only on the articular system, but also on the entire musculoskeletal, respiratory and cardio-circulatory systems. On this latter point, it would be necessary to introduce a standardised sports examination that favours a complete cardio-respiratory clinical check, which goes beyond simple chest auscultation. For this reason, the authors strongly recommend promoting educational events for dog handlers to raise their awareness of the harmful effects of work on dogs suffering from inflammatory or degenerative joint diseases and other diseases related to ‘professional’ training and on field activity.

Furthermore, 49.1% of respondents administered diet supplements to their dogs, generally represented by chondroprotective components in order to prevent articular disease in such active dogs. However, no certain preventive effect has been proven in scientific literature in healthy dogs [18]. These ‘disease-modifying’ products are generally included in canine diets with the aim to also obtain pain relief in osteoarthritic dogs; however, a recent meta-analysis has reported a weak efficacy of collagen and a very marked non-effect of chondroitin-glucosamine nutraceuticals. The meta-analysis also supported the use of omega-3 supplementation for the management of canine and feline osteoarthritis [18].

Transportation is another crucial issue for working dogs’ management, because it has been proven to be stressful and its familiarisation needs a positive early exposure in life [17]. Luckily, 86.4% of handlers and dogs usually travelled in air-conditioned cars or vans, avoiding the warmer seasons and the onset of heatstroke, a pathological condition often reported in working dogs [19].

The final sections of this survey highlighted interesting aspects of the management of the working dog: the incidence of trauma (relatively high) and prevention (extremely limited).

Injuries generally occurred in very active dogs, above all when they were employed in high-impact sports, i.e., agility or flyball. As previously described, these disciplines have reported an injury rate varying from 22% to 41% [1,3]. Our respondents reported an injury rate of ~45% on the locomotor system, but these injuries were generally represented by mild muscular trauma with a rapid resolution and absence of recurrence. Our incidence rate was almost similar to that reported by Perchette Markley et al. (2022) in a recent publication on dogs competing in agility [1], while it was above the injury report of 21.9% of endurance sports such as Canicross [20].

Canicross is a sport in which dogs generally run attached to the handler on a distance varying from 1 to 45 km or more, in a rural or wooded environment, with uneven terrain in terms of texture and slope. These conditions could hypothetically lead to an increase in injuries to handlers and dogs, but correct conditioning is probably the reason for the quite low trauma incidence in this discipline [20]. For this reason, our initial hypothesis was reasonably related to the idea that search and rescue dogs working in outdoor environments and uneven terrain might have a similar injury rate to Canicross dogs; however, the higher injury rate of our report could depend on the high number of dogs (34.5%) that attended initial training before passing the aptitude clinical exam and that had not still gained a high level of physical fitness or who had started their training course too early.

Warm-up/cool-down exercises and their real effect on injury prevention have been largely discussed in the literature, but prospective studies have still not been conducted in veterinary medicine; however, it is widely accepted that execution of specific conditioning exercises (e.g., aerobic, strengthening, or proprioceptive or balance training) may improve physical fitness [21]. In our report, only 20% of 75 respondents described a moderate pre-competition activity, with generally only a 5–10 min-walk as warm-up and a scarce or absent cool-down. It would be necessary to pay more attention to these two phases both in training or competition and specific prospective studies are absolutely necessary to understand their effectiveness in injury prevention. Apparently, most of the dogs for which trauma was reported in our survey, had performed a pre-activity warm-up; however, it should be noted that our questionnaire did not specify whether the warm-up had been planned before or after the traumatic event mentioned by the owner.

The last part of our survey was dedicated to the handler’s perception of further updating training courses to improve dog management, in order to reduce the incidence of trauma. It was positive that the open final comments by respondents were generally concentrated on a request to increase the opportunities for frequent educational courses.

A first limitation of this report could be related to the online questionnaire itself with intrinsic disadvantages such as recall bias, lack of veterinary records/verification, and selection bias. Additionally, the owners are reporting in a single time-point, thus cause/effect cannot be determined: for example, the question in the survey related to warm-up did not specify if this activity was planned from the beginning of the conditioning period of puppies or only after a trauma, therefore care should be taken to interpret the potential impact of the warm-up or cooldown.

Moreover, another limitation of this study could be also related to the moderate number of respondents (with open answers not always complete and clear): however, the percentage of trauma was high and could be due to the fact that handlers with injured dogs could be more sensitive to the topic of the study and more motivated in attending to the survey, hoping to stimulate answers on the correct management of their dogs.

## 5. Conclusions

This study, despite the limited number of cases, has highlighted the need to further promote the training of handlers and owners of working dogs to make them aware of the risks that their dogs run during their professional activity. The injury incidence was particularly high, even though the injuries reported by handlers were mostly mild with a rapid recovery. In the future, it would be useful to carry out studies aimed at the individual categories of working dogs, to better understand the impact that each specific discipline (i.e., military, IGP, avalanche, water rescue, etc.) has on dogs that practice that working activity.

## Data Availability

Not applicable. All data are reported within the text.

## Supplementary Materials

The following supporting information can be downloaded at: https://www.mdpi.com/article/10.3390/ani13101647/s1, summary pdf of English version of survey.

## References

[1] Pechette Markley A., Shoben A.B., Kieves N.R. (2021). Internet-based survey of the frequency and types of orthopedic conditions and injuries experienced by dogs competing in agility. J. Am. Vet. Med. Assoc..

[2] Tomlinson J.E., Manfredi J.M. (2018). Return to Sport after Injury: A Web-Based Survey of Owners and Handlers of Agility Dogs. Vet. Comp. Orthop. Traumatol..

[3] Cullen K.L., Dickey J.P., Bent L.R., Thomason J.J., Moëns N.M.M. (2013). Survey-based analysis of risk factors for injury among dogs participating in agility training and competition events. J. Am. Vet. Med. Assoc..

[4] Cullen K.L., Dickey J.P., Bent L.R., Thomason J.J., Moëns N.M.M. (2013). Internet-based survey of the nature and perceived causes of injury to dogs participating in agility training and competition events. J. Am. Vet. Med. Assoc..

[5] Essner A., Hesbach A.L., Igelström H., Kjellerstedt C., Svensson K., Westerlind H. (2022). Physical activity and sport-specific training patterns in Swedish sporting and working trial dogs—A questionnaire survey. Front. Vet. Sci..

[6] Steiss J.E. (2002). Muscle disorders and rehabilitation in canine athletes. Vet. Clin. N. Am. Small Anim. Pract..

[7] Fradkin A.J., Gabbe B.J., Cameron P.A. (2006). Does warming up prevent injury in sport? The evidence from randomized controlled trials?. J. Sci. Med. Sport.

[8] Koh R., Montalbano C., Gamble L.J., Walden K., Rouse J., Liu C.C., Wakshlag L.G., Wakshlag J.J. (2020). Internet survey of feeding, dietary supplement, and rehabilitative medical management use in flyball dogs. Can. Vet. J..

[9] Pinto K.R., Chicoine A.L., Romano L.S., Otto S.J.G. (2021). An Internet survey of risk factors for injury in North American dogs competing in flyball. Can. Vet. J..

[10] Montalbano C., Gamble L.J., Walden K., Rouse J., Mann S., Sack D., Wakshlag L.G., Shmalberg J.W., Wakshlag J.J. (2019). Internet Survey of Participant Demographics and Risk Factors for Injury in Flyball Dogs. Front. Vet. Sci..

[11] Spinella G., Tidu L., Grassato L., Musella V., Matarazzo M., Valentini S. (2022). Military Working Dogs Operating in Afghanistan Theater: Comparison between Pre- and Post-Mission Blood Analyses to Monitor Physical Fitness and Training. Animals.

[12] Edwards T.H., Scott L.L.F., Gonyeau K.E., Howard E.H., Parker J.S., Hall K. (2021). Comparison of trauma sustained by civilian dogs and deployed military working dogs. J. Vet. Emerg. Crit. Care.

[13] Lagutchik M., Baker J., Balser J., Burghardt W., Enroth M., Flournoy S., Giles J., Grimm P., Hiniker J., Johnson J. (2018). Trauma Management of Military Working Dogs. Mil. Med..

[14] Evans R.I., Herbold J.R., Bradshaw B.S., Moore G.E. (2007). Causes for discharge of military working dogs from service: 268 cases (2000–2004). J. Am. Vet. Med. Assoc..

[15] Hart B.L., Hart L.A., Thigpen A.P., Willits N.H. (2014). Long-term health effects of neutering dogs: Comparison of Labrador Retrievers with Golden Retrievers. PLoS ONE.

[16] Sundby A.E., Pechette Markley A., Shoben A.B., Kieves N.R. (2022). Internet Survey Evaluation of Demographic Risk Factors for Injury in Canine Agility Athletes. Front. Vet. Sci..

[17] Cobb M.L., Otto C.M., Fine A.H. (2021). The Animal Welfare Science of Working Dogs: Current Perspectives on Recent Advances and Future Directions. Front. Vet. Sci..

[18] Barbeau-Grégoire M., Otis C., Cournoyer A., Moreau M., Lussier B., Troncy E. (2022). A 2022 Systematic Review and Meta-Analysis of Enriched Therapeutic Diets and Nutraceuticals in Canine and Feline Osteoarthritis. Int. J. Mol. Sci..

[19] Carter A.J., Hall E.J., Connolly S.L., Russell Z.F., Mitchell K. (2020). Drugs, dogs, and driving: The potential for year-round thermal stress in UK vehicles. Open Vet. J..

[20] Lafuente P., Whyle C. (2018). A Retrospective Survey of Injuries Occurring in Dogs and Handlers Participating in Canicross. Vet. Comp. Orthop. Traumatol..

[21] Evanow J.A., Van Deventer G., Dinallo G., Mann S., Frye C.W., Wakshlag J.J. (2021). Internet Survey of Participant Demographics and Risk Factors for Injury in Competitive Agility Dogs. VCOT Open.

