# Supplementary material for: Internet-Based Survey on Physical Activity and Incidence of Injury in Active Working Dogs
Source: Animals (Basel). 2023 May 16;13(10):1647. doi: 10.3390/ani13101647 (PMC10215327; doi:10.3390/ani13101647)
Supplement: Supplementary file 1 [file animals-13-01647-s001.zip › animals-2357790-supplementary.pdf]

SURVEY ON WORKING DOGS

(TRASLATION FROM ITALIAN FORM)

SECTION 1

- CANINE BREED_______________________

- CANINE SEX STATUS:
  - Neutered/Spayed
  - Intact/Breeding

- AGE

- DEWORMING FREQUENCY
  - only as a puppy
  - sporadically
  - once a month
  - once a year
- VACCINE SCHEDULE
  - only as a puppy
  - sporadically
  - once a year
- HEARTWORM PREVENTION
  - never
  - sporadically
  - once a month
  - once a year
- TREATMENT FOR HEARTWORM PREVENTION
  - tablets
  - vaccination
  - spot on
  - other
- LEISHMANIASIS PREVENTION
  - never
  - sporadically
  - once a month
  - once a year
- TREATMENT FOR LEISHMANIASIS PREVENTION
  - collar
  - vaccination
  - spot on
- CONTROL OF EXTERNAL PARASITES
  - never
  - sporadically
  - once a month
  - once a year
- TREATMENT FOR EXTERNAL PARASITES PREVENTION
  - collar

  - tablets
  - spot on
  - other
- HAS YOUR DOG UNDERGONE AN EARLY X-RAY EXAMINATION?
  - Yes
  - No
- IF YES, SPECIFY AT WHAT AGE(MONTHS/YEARS)
- IF YES, SPECIFY WHICH ANATOMICAL REGIONS WERE EXAMINED
- HAS YOUR DOG UNDERGONE A CONFIRMATORY X-RAY EXAMINATION?
  - Yes
  - No
- IF YES, SPECIFY AT WHAT AGE (MONTHS/YEARS)
- IF YES, SPECIFY WHICH ANATOMICAL REGION/S WAS/WERE EXAMINED
- HAS YOUR DOG UNDERGONE AN ORTHOPEDIC CHECK?
  - Yes
  - No
- IF YES, SPECIFY AT WHAT AGE (MONTHS/YEARS)
- IF YES, SPECIFY WHY
- HAS YOUR DOG UNDERGONE A SPORTS MEDICAL EXAMINATION?
  - Yes
  - No
- IF YES, SPECIFY AT WHAT AGE (MONTHS/YEARS)
- IF YES, SPECIFY WHY
- HAD YOUR DOG UNDERGONE GENERAL BLOOD TESTS?
  - Yes
  - No

  - never
  - only as a puppy
  - sporadically
  - once a year
- IF YES, SPECIFY WHY
- IF YES, SPECIFY WHICH TEST
- HAS YOUR DOG UNDERGONE A CARDIOLOGICAL EXAMINATION?
  - never
  - only as a puppy
  - sporadically
  - once a month
  - once a year

- IF YES, SPECIFY WHY
- DO YOU KNOW CANINE PHYSIOLOGICAL PARAMETERS? (RESPIRATORY RATE, HEART RATE, TEMPERATURE)
  - Yes
  - No
- IF YES, PLEASE REPORT THE PHYSIOLOGICAL VALUES

SECTION 2

- DESCRIBE YOUR DOG’S DIET
    - BARF
    - cooked meat
    - dry food
    - canned food
    - other
- HOW OFTEN DO YOU FEED YOUR DOG?
- DO YOU OFFER FOOD BEFORE TRAINING? (SPECIFY HOW LONG BEFORE WORK THE DOG REMAINS FASTING)
- DO YOU MAKE ANY DIETARY CHANGES THROUGHOUT THE YEAR?
    - Yes
    - No
- IF YES, SPECIFY WHY

SECTION 3

- ARE DIETARY SUPPLEMENTS PROVIDED?
    - Yes
    - No
- IF YES, SPECIFY WHICH ONES AND THE FREQUENCY OF ADMINISTRATION
- ARE CHONDROPROTECTORS PROVIDED?
    - Yes
    - No
- IF YES, SPECIFY WHICH ONES AND THE FREQUENCY OF ADMINISTRATION
- ARE ANY DRUGS GENERALLY PROVIDED?
    - Yes
    - No
- IF YES, SPECIFY WHICH ONES AND RATE OF ADMINISTRATION

SECTION 4

- WHERE DOES THE DOG USUALLY LIVE?
    - Owner’s home
    - Owner’s home and garden
    - Owner’s home garden
    - Other place than the owner's home
- IF THE DOG LIVES IN THE GARDEN, SPECIFY WHETHER HE/SHE IS…
    - Free
    - In a dog fence
- WHAT IS THE TYPE OF SURFACE (GROUND, GRAVEL, GRASS…) ON WHICH YOUR DOG MAINLY LIVES?
- WHERE DOES THE DOG SLEEP ON?
    - Kennel
    - Dog bed
    - Ground
    - Other

SECTION 5

- HOW THE DOG IS TRANSPORTED (CAR, VAN, TRAILER, OTHER …)?
- DURING TRANSPORT THE DOG IS …
    - Unrestrained
    - In cage
    - Other
- IS THE ENVIRONMENT WHERE THE DOG IS LOCATED DURING TRANSPORT AIR-CONDITIONED?
- SPECIFY THE MINIMUM AND MAXIMUM TRANSPORTING TIME
- IS YOUR DOG TRANSPORTED IN OTHER VEHICLES FOR WORKING? (HELICOPTER, CABLE CAR, BOAT)
    - Yes
    - No
- IF YES, SPECIFY WHICH ONE

SECTION 6

- HOW DO YOU USUALLY RESTRAIN YOUR DOG?
    - Collar
    - Harness
    - Other
- SPECIFY THE TYPE OF COLLAR OR HARNESS
- DURING TRAINING THE DOG IS LED:
    - On a leash
    - Unrestrained
    - Other
- SPECIFY THE LEASH LENGTH
- WHEN WORKING, THE DOG WEARS OTHER TYPES OF HARNESS?
- IF YES, SPECIFY WHIC ONE (FOR EXAMPLE, AIR AMBULANCE)
- IF YOUR DOG WEARS SPECIFIC HARNESS (FOR EXAMPLE HELICOPTER HARNESS) DURING WORK THE HARNESS IS REMOVED OR NOT?
- WHEN THE DOG STARTS WORKING AFTER REMOVING THE HARNESS, DO YOU NOTICE THE DIFFERENCE IN MOVEMENTS/GAIT?

SECTION 7

- WHAT IS YOUR DOG’S WORK?
- IS YOUR DOG’S WORK …?
    - A part-time work (hobby)
    - A full-time work
- AT WHAT AGE DID THE DOG START WORKING?
- HOW MANY DAYS A WEEK DOES THE DOG WORK?
- IS YOUR DOG … ?
    - In training
    - In activity
    - Initial level
- WHAT IS THE SURFACE (GROUND, RUBBLE, WATER…) WHERE YOUR DOG MAINLY WORKS?

SECTION 8

- WHAT DO YOU MEAN FOR “TRAINING/CONDITIONING”?
- WHAT IS YOUR DOG’S TYPE OF TRAINING?
- HOW MANY DAYS A WEEK IS YOUR DOG TRAINED?
- IS THE TRAINING SPECIFIC FOR THE WORK PERFORMED BY THE DOG?

SECTION 9

- DO YOU APPLY ANY PREVENTION OF INJURIES FOR YOUR DOG IN WORKING/TRAINING CONDITIONS?
    - No prevention
    - Prevention
- DESCRIBE THE INJURY PREVENTION PROTOCOL (MASSAGE, ETC)
- BEFORE WORKING, DOES THE DOG WARM UP? IF YES, FOR HOW LONG AND WHAT KIND OF EXERCISES ARE PERFORMED (DESCRIBE)
- AFTER WORKING, DOES THE DOG COOL DOWN? IF YES, FOR HOW LONG AND WHAT KIND OF EXERCISES ARE PERFORMED (DESCRIBE)
- WHO HAS DRAFTED THE PREVENTION PROTOCOL?
    - The veterinarian
    - Physiatrist vet
    - Coach
    - By myself
    - Other
- DESCRIBE HOW YOU PREVENT INJURIES ON THE LIMBS (FRONT AND REAR)
- DESCRIBE HOW YOU PREVENT INJURIES ON THE SPINE
- HOW MUCH TIME DO YOU DEDICATE TO PREVENTION (SPECIFY MINUTES AND FREQUENCY)?
- DESCRIBE HOW YOU PREVENT INJURIES AFTER WORK
    - No prevention
    - Massage
    - Warm compresses
    - Other

SECTION 10

- IN YOUR OPINION, WHAT ARE THE PAIN SIGNALS THAT LET YOU UNDERSTAND THE PRESENCE OF AN INJURY?
- HAS YOUR DOG EVER BEEN INJURED?
    - Yes
    - No
- IF YES, DESCRIBE THE INJURY
- HOW MANY INJURIES (TRAUMA TO THE MUSCULOSKELETAL SYSTEM) HAS YOUR DOG SUFFERED?
- DID THE EFFECTS OF THE TRAUMA SHOW UP IMMEDIATELY DURING/AFTER WORK OR IN THE FOLLOWING DAYS?
- DID YOUR DOG'S INJURY PRODUCE SHORT-TERM OR LONG-TERM SIGNS?
- SPECIFY HOW LONG THE DOG HAS BEEN AT REST
- HAS THE TRAUMA ALWAYS AFFECTED THE SAME LIMB? IF SO, WHICH ONE ?
- HAS THE INJURY RECURRED?
- WHO DIAGNOSED THE INJURY?

- A veterinarian
    - Coach
    - Owner him/herself
    - Other
- WHO PROVIDED INJURY THERAPY?
    - A veterinarian
    - Coach
    - Owner him/herself
    - Other
- HAVE YOUR DOG EVER NEEDED REHABILITATION TREATMENT AFTER AN INJURY?
    - Yes
    - No
- IF YES, SPECIFY WHICH REHABILITATION TREATMENT
- HAVE YOUR DOG EVER NEEDED SURGERY AFTER AN INJURY?
    - Yes
    - No
- IF YES, SPECIFY WHICH SURGERY
- IF YOUR DOG HAS SUFFERED MULTIPLE INJURIES, SPECIFY WHERE, HOW AND IF INJURIES RESOLVED DEFINITIVELY

SECTION 11

- DID YOU ATTEND COURSES ON THE WELL-BEING OF YOUR DOG?
    - Yes
    - No
- IF YES, SPECIFY WHICH COURSE
- WOULD YOU AGREE TO MAKE CANINE SPORTS VISIT ANNUALLY MANDATORY?
    - Yes
    - No
- WHY?
- DO YOU HAVE ANY SUGGESTIONS OR WOULD YOU LIKE TO EXPRESS YOUR OPINION ON THE WELFARE OF WORKING DOGS?

SECTION 12

I AUTHORIZE THE USE OF THE AGGREGATED DATA IN AN ANONYMOUS FORM FOR EPIDEMIOLOGICAL STUDIES AND FOR ANY SCIENTIFIC DISCLOSURES AT SCIENTIFIC MEETINGS AND/OR JOURNALS

- Yes
- No

SECTION 13

PERSONAL COMMENTS OR SUGGESTIONS ON WORKING DOGS’ WELFARE
